# “It’s Good to Have These Conversations”: An Exploration of the Views of Psychiatrists on the Use of Alternatives to Psychiatric Diagnoses

**DOI:** 10.1192/bjo.2026.11061

**Published:** 2026-06-30

**Authors:** Jodie Mairs, Declan Hyland

**Affiliations:** Mersey Care NHS Foundation Trust, Liverpool, United Kingdom

## Abstract

**Aims:**

The value and utility of psychiatric diagnoses have been heavily debated within the literature, with proponents advocating that they offer evidence-based treatment and ease of professional communication. The role of social determinants of mental health has been gaining traction, and one alternative to psychiatric diagnoses has been proposed to use phenomenological codes within the current medical system to record both symptoms and the social determinants that service users report. The literature suggests that this approach has been minimally used, however, to date, no research has explored mental health professionals’ views on using this within clinical practice. This research had two broad aims: to elicit the views of psychiatrists working within general adult mental health services on the use of psychiatric diagnoses and alternatives to these, with a focus on using phenomenological codes and to explore how these alternatives may be utilised within clinical practice, specifically focusing on the positives and challenges.

**Methods:**

14 Consultant Psychiatrists were recruited from three NHS sites across the North West of England from a range of adult mental health services. Semi-structured interviews were completed either virtually or face-to-face which included a video explaining the idea of using phenomenological codes. The interviews were recorded with consent, transcribed, and analysed using reflexive thematic analysis.

**Results:**

Six overarching themes and 18 subthemes were generated from the interviews. These included: *psychiatric diagnoses are imperfect but practical, coding may be helpful but impractical, we are all just stuck in the system, psychological input is helpful but a luxury* and *moving forwards towards best practice.* The results demonstrated the multitude of factors that both impacted on views of psychiatric diagnoses and the ability to implement potential alternatives within NHS mental health services.

**Conclusion:**

Overall, participants demonstrated enthusiasm towards non-diagnostic approaches and exploring the role of social determinants, however felt that coding would be logistically challenging. Participants discussed the complexity of this area, exploring both the impact of personal and professional views shaped by experience alongside wider systemic factors such as professional identity and the constraints within NHS services. Future research could explore further how to integrate medical and non-diagnostic approaches to highlight the role of social determinants in the development of mental health difficulties and how this can be effectively applied within clinical services.

